# Selective Electrical Surface Stimulation to Support Functional Recovery in the Early Phase After Unilateral Acute Facial Nerve or Vocal Fold Paralysis

**DOI:** 10.3389/fneur.2022.869900

**Published:** 2022-04-04

**Authors:** Annabella Kurz, Gerd Fabian Volk, Dirk Arnold, Berit Schneider-Stickler, Winfried Mayr, Orlando Guntinas-Lichius

**Affiliations:** ^1^Department of Otorhinolaryngology, Division of Phoniatrics-Logopedics, Medical University of Vienna, Vienna, Austria; ^2^Department of Otorhinolaryngology, Jena University Hospital, Jena, Germany; ^3^Facial-Nerve-Center Jena, Jena University Hospital, Jena, Germany; ^4^Center for Rare Diseases, Jena University Hospital, Jena, Germany; ^5^Center for Medical Physics and Biomedical Engineering, Medical University of Vienna, Vienna, Austria

**Keywords:** electrical stimulation, facial nerve, vagal nerve, recurrent laryngeal nerve, nerve stimulation, muscle stimulation, nerve regeneration electrical stimulation, nerve regeneration

## Abstract

This article addresses the potential clinical value of surface electrical stimulation in the acute phase of denervation after the onset of facial nerve or recurrent laryngeal nerve paralysis. These two nerve lesions are the most frequent head and neck nerve lesions. In this review, we will work out several similarities concerning the pathophysiology features and the clinical scenario between both nerve lesions, which allow to develop some general rules for surface electrical stimulation applicable for both nerve lesions. The focus is on electrical stimulation in the phase between denervation and reinnervation of the target muscles. The aim of electrostimulation in this phase of denervation is to bridge the time until reinnervation is complete and to maintain facial or laryngeal function. In this phase, electrostimulation has to stimulate directly the denervated muscles, i.e. muscle stimulation and not nerve stimulation. There is preliminary data that early electrostimulation might also improve the functional outcome. Because there are still caveats against the use of electrostimulation, the neurophysiology of denervated facial and laryngeal muscles in comparison to innervated muscles is explained in detail. This is necessary to understand why the negative results published in several studies that used stimulation parameters are not suitable for denervated muscle fibers. Juxtaposed are studies using parameters adapted for the stimulation of denervated facial or laryngeal muscles. These studies used standardized outcome measure and show that an effective and tolerable electrostimulation of facial and laryngeal muscles without side effects in the early phase after onset of the lesions is feasible, does not hinder nerve regeneration and might even be able to improve the functional outcome. This has now to be proven in larger controlled trials. In our view, surface electrical stimulation has an unexploited potential to enrich the early therapy concepts for patients with unilateral facial or vocal fold paralysis.

## Introduction

Facial and recurrent laryngeal nerve (recurrent nerve) lesion are the most common cranial nerve lesions. Like for any other peripheral nerve injury, lesions of the facial or recurrent laryngeal nerve are mainly caused by inflammation, external trauma including iatrogenic lesions after stretching, tearing, or extrusion of the nerve, tumor infiltration or are of idiopathic origin (1, 2). Classically, nerve injuries are divided into three pathophysiological categories by Seddon (3). Neurapraxia, the first-degree injury, is the most common response to a blunt trauma. It causes a temporary conduction block. The myelin sheath of the nerve fibers at the site of injury but not the axons are damaged. This leads only to temporary functional changes. Idiopathic facial palsy mostly is a neurapraxic lesion, which explains the high recovery rate under drug treatment (4). Stretch lesions of the recurrent nerve are another example (5). Axonotmesis is the second-degree lesion and refers to loss of axonal continuity without associated disruption of the fascicular connective tissue elements. This is seen in severe compression (crush) lesions. The axon and its myelin sheath are broken, yet the surrounding connective tissue framework (i.e. the endoneurium, perineurium and epineurium) remain partially or fully intact. In neurotmesis, the third-degree and the most severe nerve injury, the nerve is physically divided. This is for instance the case after intended resection of a tumor-infiltrated nerve during tumor surgery or unintended as iatrogenic lesion during any kind of head and neck surgery. If feasible, reconstruction of the lesioned facial or recurrent nerve can lead to functional recovery but also with synkinetic reinnervation (6, 7).

Since the 2000s, when first animal experiments and later first human trials have shown that electrical stimulation can accelerate peripheral nerve regeneration, there has been an increasing interest in using electrical stimulation for improving functional recovery after peripheral nerve injury (8). Denervated muscle fibers atrophy prior to their reinnervation and delayed nerve repair influence the full recovery of the size of denervated muscle fibers (9). A healthy muscle fiber is activated by the natural electrical potentials transferred to the muscle *via* the innervating nerve. Electrical signals of appropriate speed of change of the intensity per time (like 0.5 mA per 0.2 ms) are generating the same electrical potentials in the innervating nerve. Therefore, electrical stimulation evokes the same activation of the innervated muscle fibers. This review focusses electrostimulation of the denervated muscles, hence to use electrostimulation for direct muscle stimulation: A denervated muscle does not receive innervation and therefore needs to be activated by electrical signals applied to the muscle fibers directly. The electrical amplitude change required for that is about 10–20 times higher than for innervated muscles, and the same is for the required duration of the electrical pulse (like 5 mA per 2 ms). Stimulating denervated muscle fibers via surface electrodes requires an additional increase of amplitude and pulse duration of the electrical signal (like 10–15 mA per 20–5 0 ms), since the tissue in-between the electrode and muscle is filtering out a big percentage of the signal. Over many years, the effects of electrical stimulation showed controversial results in animal experiments (10, 11). Today, we know that timing and the stimulation parameters are crucial to ensure positive effects. Based on this, human studies have clearly shown that electrical stimulation of denervated muscles can retain and even regain muscle (12). Due to the high demand, most electrical stimulation research in the head and neck region has been performed on the facial nerve and the recurrent nerve.

In the recent years, more and more clinical studies build evidence that electrical stimulation is a suitable clinical treatment in the acute phase of acute facial or recurrent nerve lesion. We aim to review the positive effects of the latest advances in electrical stimulation during the early phase of a facial or recurrent nerve lesion on muscle atrophy prevention and preservation of specific muscle function. Hereby, we want to pay attention to the common features of electrical stimulation to restore facial and recurrent nerve function in the phase from onset of denervation to regeneration of the target muscles. The focus will be on this efferent function, being aware that the peripheral facial nerve is distal to its exit of the stylomastoid foramen a pure motor nerve whereas the recurrent nerve is a mixed nerve. This review will focus on efferent electrical stimulation. This review will not touch upon electrical stimulation of chronic, long-term denervated facial or laryngeal muscles.

To provide a comprehensive overview on these topics, a systematic literature search was conducted using the PubMed and ScienceDirect database. The search included all articles in English and German language published from database inception to December 31, 2021. Keywords and MeSH terms included: “facial nerve,” “recurrent nerve,” “recurrent laryngeal nerve,” “Bell palsy,” “vocal fold paresis,” “vocal fold paralysis,” “facial nerve paresis,” “facial nerve paralysis,” “facial nerve diseases,” “facial muscle denervation,” “laryngeal muscle denervation,” “electrodiagnostics,” “electromyography,” “outcome,” “clinical study,” and “humans.” All studies investigating acute facial or recurrent laryngeal nerve lesion without denervation (neurapraxia), or studies for electrostimulation after muscle reinnervation were excluded. Due to the lack of high-quality clinical trials or even of larger observational studies, case reports were also included.

## Facial or Recurrent Laryngeal Nerve Lesion and Time Course of Nerve Regeneration, Muscle Atrophy and Muscle Reinnervation

### Facial Nerve Lesion, Regeneration, and Facial Muscle Reinnervation

The most common facial nerve injury is the idiopathic facial palsy (Bell's palsy). Most idiopathic cases are neurapraxic lesions. About about 64% of idiopathic facial palsy cases recover in 3 months, and other 31% between 9 to 12 months when treated under the standard corticosteroid therapy (13, 14). Recovery is here defined as the full recovery of facial movement as measured by facial grading systems. In contrast, the recovery rates are lower in case of axonal injury (axonotmesis) with 30, 40, and 40% at 3, 9, and 12 months (15).

Unless the regeneration over the neurotmetic lesion is not hindered, a transected facial nerve can regenerate and reinnervate their target organs, e.g. the mimic muscles. The axon regrowth depends on the synthesis and transportation of the intracellular substances. The regeneration speed is therefore about 1–3 mm/day (16, 17). Before that, Wallerian degeneration of the facial nerve distal to the lesion site has to take place. The Wallerian degeneration needs 10–4 days to reach the facial muscles as it can be seen by the occurrence of pathological spontaneous activity in the electromyographic (EMG) examination (6). If the degeneration is not further progressive (like it could be in case of a tumor infiltration), the denervation of the facial muscles is complete within 2- months after onset (18). After 6 months, most patients with spontaneous regeneration after neurotmesis or regeneration induced by facial nerve repair show regeneration potentials in the facial muscles (19). Muscle regeneration is completed after 12–15 months of reinnervation. The effects of denervation and reinnervation on the facial muscles, muscle volume and muscle contractibility are unknown. A denervated muscle progressively loses its ability to become reinnervated (17, 20). The process of muscle atrophy seems to be very variable (21). It seems that facial muscle degeneration is very slow as electrical stimulation of denervated facial muscles is possible even after years (22).

After neurotmesis, all patients show at least some degree of facial synkinesis, i.e. the occurrence of involuntary movement of the facial mimetic musculature accompanying voluntary facial movements (20). The facial nerve innervates many mimic muscles with different functions. The muscles partly even behave agonistic and antagonistic during specific tasks. Therefore, synkinesis as a coordination disorder plays an important role in the chronic phase of facial palsy. The time course is identical, whether after spontaneous reinnervation or after facial nerve reconstruction. First synkinetic activity after intratemporal or main trunk lesion occurs after 5–6 months and the final pattern is reached about 12 months after onset of the lesion or nerve repair (18, 23).

### Recurrent Nerve Lesion, Regeneration, and Laryngeal Muscle Reinnervation

In contrast to the peripheral facial nerve, the recurrent nerve is a mixed nerve carrying motor, sensory, and secretory fibers (24). Here, we focus only on the regeneration of the motor fibers. The spectrum of etiologies and pathophysiology is comparable to the facial nerve. Iatrogenic trauma induced by thyroid surgery is the most common etiology of vocal fold paralysis (25). The immobility or hypomotility of the vocal folds is not the obvious and most important symptom, but rather the resulting dysphonia. This is crucial to keep in mind when analyzing the data regarding the outcome measure. Functional rehabilitation in terms of improved glottic competence through restoration of muscle mass and tone after reinnervation may occur even in the absence of physiologic motion recovery (26). Concerning the recovery rate, reliable data has only been published on iatrogenic vocal fold paralysis. Up to 90% of the cases with unilateral vocal fold paralysis show a recovered vocal function (26). In contrast, only 23–75% of the patients show a return of motion (26–28). Whereas mimic movement analysis is clear, the wide range for vocal fold movement analysis is explained by inconsistent definitions of return of motion (26). The presumed regeneration speed is with 1–3 mm/day the same as for the facial nerve (26). In case of mild axonotmesis, EMG signs of reinnervation are seen already at 2 months after onset. Recovery will take on average 5–6 months and is complete at the latest after about 12 months (26–29).

In case of axonal injury, signs of denervation in the laryngeal musculature can be seen up to 4 months (average: about 2 months) (29, 30). Like the facial nerve, the recurrent nerve drives a complex set of muscles with agonistic and antagonistic function. Therefore, synkinesis plays also an important role as long-term sequela for patients with recurrent nerve lesions. Synkinetic activity is first seen after 2 months depending on the location of the lesion (29). Whereas synchronous EMG of several facial muscles is standard to verify facial synkinesis, this is not routinely performed for laryngeal EMG. This is important, since the patterns of synkinetic reinnervation in the larynx seem to be etiology-dependent. The rates of synkinetic activity in the posterior cricoarytenoid (PCA) muscle in comparison to the thyroarytenoid (TA) muscle are different dependent on the etiology (31), and the laryngoscopic classification might be different from the EMG classification (32). As it is the case for facial nerve injuries, not much is known about the time course of the laryngeal muscle atrophy. Based on computed tomography analysis, it has been shown that within 1 month after the lesion, some degree of atrophy of the PCA muscle appears in >90% of the cases, and severe atrophy already in 13% of the cases (33). In case of permanent immobility, the degree of atrophy correlates with the reinnervation status. The pattern of persistent denervation and synkinetic reinnervation seems to be variable with a persistent partial denervation rate of 42 and 27% in the PCA and the TA, respectively. What makes the situation even more complex is that former inspiratory slow-twitch axons (originally innervating the PCA) may reinnervate the TA (originally innervated by fast-twitch fibers). This may result in a medial bulging of the vocal cord during inspiration, a phenomenon that may produce glottic airway obstruction (34). The muscle twitch characteristics have influence on the electrical stimulation parameters. Fast twitch muscles like the normal TA show tetanic contractions at higher fusion frequencies (35). Smooth contraction can be expected only by stimuli with a frequency equal or higher than the muscle fusion frequency (36). Hence, any change of the fiber-type composition after nerve injury and pathological regeneration can have influence on the stimulation parameters.

## The Goals of Electrical Stimulation are Different in the Acute Phase of Denervation After Nerve Lesion

It is important to consider the phase during which electrical stimulation should be applied (Table 1). Electrical stimulation has different possibilities and limitations, either early on, when the target muscles are denervated, or later during reinnervation when the muscles can already be partly reinnervated but still are partly denervated. The scenario again is very different in the chronic phase: here the muscles can be either chronically denervated without any remaining viable nerve fibers within the musculature, or the muscles can be synkinetically reinnervated. This all is very important, because electrical stimulation of the (non-regenerating or regenerating) facial or recurrent nerve to excite the target muscles, i.e. nerve stimulation, needs different stimulation parameters than direct stimulation of the denervated muscles, i.e. muscle stimulation. Partly, negative results of studies on electrical stimulation for patients with facial or recurrent nerve lesion can quite simply be explained by the application of non-suitable stimulation parameters. This review will focus on electrical stimulation in the early phase after nerve lesion in which the nerve retains the capability to regenerate and the program of reinnervation of the target muscles is initiated.

**Table 1:** Potential scenarios for electrical stimulation (ES) after facial or recurrent nerve lesion.

**Phase of ES**	**Status of the lesioned nerve**	**Status of the target muscles**
In the acute phase of nerve lesion and denervation		
Intraoperative ES	Freshly lesioned nerve	Muscle status unchanged
Early onset ES	Nerve axons start to regenerate, muscles are not reinnervated yet	Muscles are denervated
ES during reinnervation	Some axons have reinnervated target muscles, reinnervation ongoing	Muscle partly denervated, partly reinnervated
In the chronic phase of nerve lesion and denervation or reinnervation		
ES for flaccid paralysis	No viable nerve	Denervated muscles with progressive atrophy
ES for post-paralytic synkinesis	Pathological reinnervation terminated	Muscles are synkinetically reinnervated

*This review is focused on ES early after onset of the lesion and muscle denervation up to the phase of muscle reinnervation (highlighted in gray)*.

The temporal scenarios are linked to the functional types and goals of electrical stimulation (Table 2). Electrical stimulation can be separated into invasive and non-invasive. Invasive stimulation means as a direct application of the electrical stimulus on the exposed facial or recurrent nerve or muscle. Such a situation is only given during nerve reconstruction surgery, when electrical stimulation can be applied directly on the reconstructed nerve, or via needles inserted next to the stimulation target. This has not been tested for the facial or recurrent nerve in humans (37). This limit could only be overcome by implantable systems. Electrical peripheral nerve stimulation with implantable devices is established to treat chronic pain (38), but not for acute motor nerve stimulation. First phase 1 trials with a recurrent laryngeal nerve stimulator have only been performed for patients with chronic bilateral vocal fold immobility in combination with synkinetic reinnervation (39). Instead, most frequently, non-invasive transcutaneous nerve stimulation is used. The consequence is that the stimulation parameters have to be chosen in such a way that stimulation of other structures between the surface electrode and the target nerve (like pain fibers or other nerves and muscles) has to be avoided. In contrast to nerve stimulation, muscle stimulation of the denervated target muscles again needs significantly different stimulation parameters. This type of stimulation aim to cause contractions that prevent or slow down atrophy, as well as, potentially support the regeneration of peripheral nerves.

**Table 2:** Definition of different types of electrical stimulation (ES) with different therapy goals or different invasiveness.

**Term**	**Definition**
Functional ES	The stimulation by ES triggers coordinated contractions to support restricted or absent motor function.
Neuromuscular ES	Here, ES is a more passive treatment approach. Predominantly structural and functional deficits stand in the foreground
Bridging ES	ES is bridging the period between the first onset of the facial or recurrent laryngeal nerve lesion and muscle-preserving surgery, to prevent atrophy of mimic or laryngeal muscles
Conditioning ES	ES should promote nerve regeneration back to the original target and decrease misdirected reinnervation
Invasive ES	Direct ES on the nerve and/or muscle, needs invasive exposure of the nerve and/or muscle
Non-invasive ES	Transcutaneous ES without opening of the body surface

In summary, theoretically, the goals of electrical stimulation early after acute facial nerve or vocal fold paralysis can be to: a) prevent muscle atrophy, b) maintain muscle function during the phase of denervation and reinnervation, c) accelerate the nerve regeneration and hence the recovery, d) increase preferential nerve sprouting, misdirected reinnervation leading to synkinesis, e) prevent loss of cortical representation of original movement patters, and f) to improve to improve the functional outcome. This would at best lead to restoration of mimic function after facial palsy, or to optimal voice recovery after recurrent laryngeal nerve lesion without side effects. For optimal evaluation of the functional outcome in the face, the outcome analysis should at best include a standardized grading of the mimic function, an electrophysiological assessment of the facial nerve and muscles, and facial-specific patient-recorded outcome measures (facial PROMs) (6, 40, 41). For optimal evaluation of laryngeal recovery, this means a laryngoscopic evaluation, laryngeal electrophysiology, a voice analysis, and voice/breathing-related PROMs (42–45).

## Principles of Selective Stimulation of a Denervated Muscle

The design of an optimal muscle stimulation must consider that non-selective stimulation of pain fibers and neighboring muscles/tissue between the stimulation site and the targeted muscles is the main limiting factor for the selection of stimulation parameters. On the other hand, the amplitude needed for functional activation of denervated muscle fibers is 10–100 times higher than the amplitude needed for activation of nerve fibers. Hence, it is much more difficult to selectively stimulate a denervated muscle without side effects (22, 26, 36, 37).

**Figure 1:**
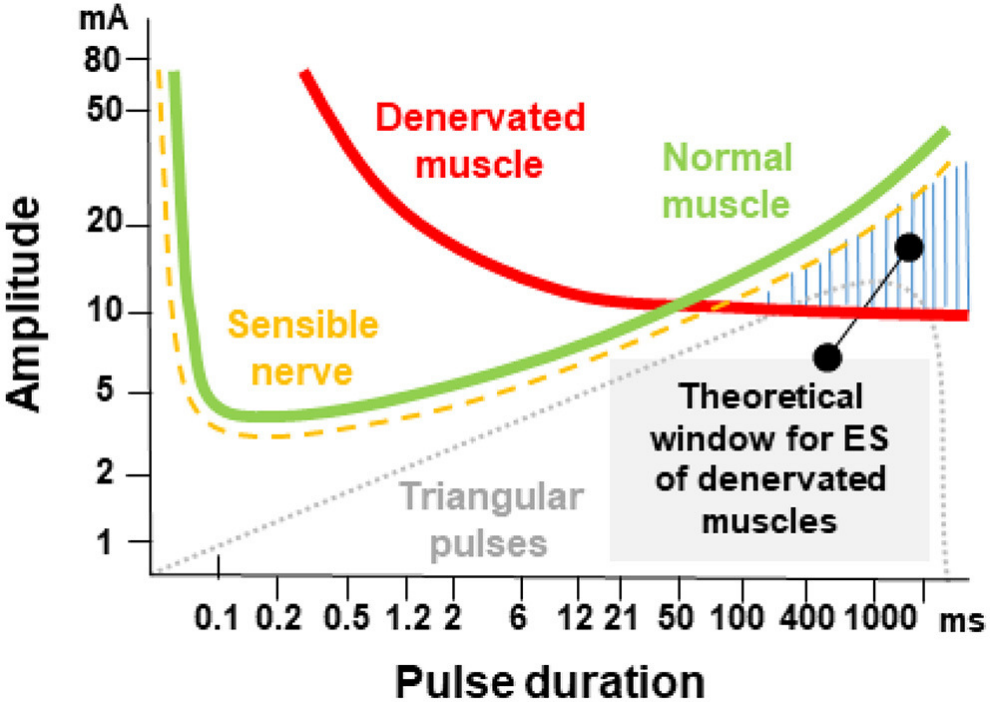
Pulse duration = rise time in ms versus threshold current in mA (I/T curves). The curves for normal innervated muscle (green) and denervated muscles (red) cross at 50–100 ms pulse duration. The damaged muscle responds to longer triangular pulses even at lower currents than the normal muscle or the sensitive nervous elements of the skin. The threshold curve of the sensitive and pain fibers run below the threshold curve of normal muscle fibers green. The denervated muscle can be selectively stimulated with long triangular pulses in the hatched area. The original concept was developed by Thom (46) and Martin and Witt (47).

**Figure 2:**
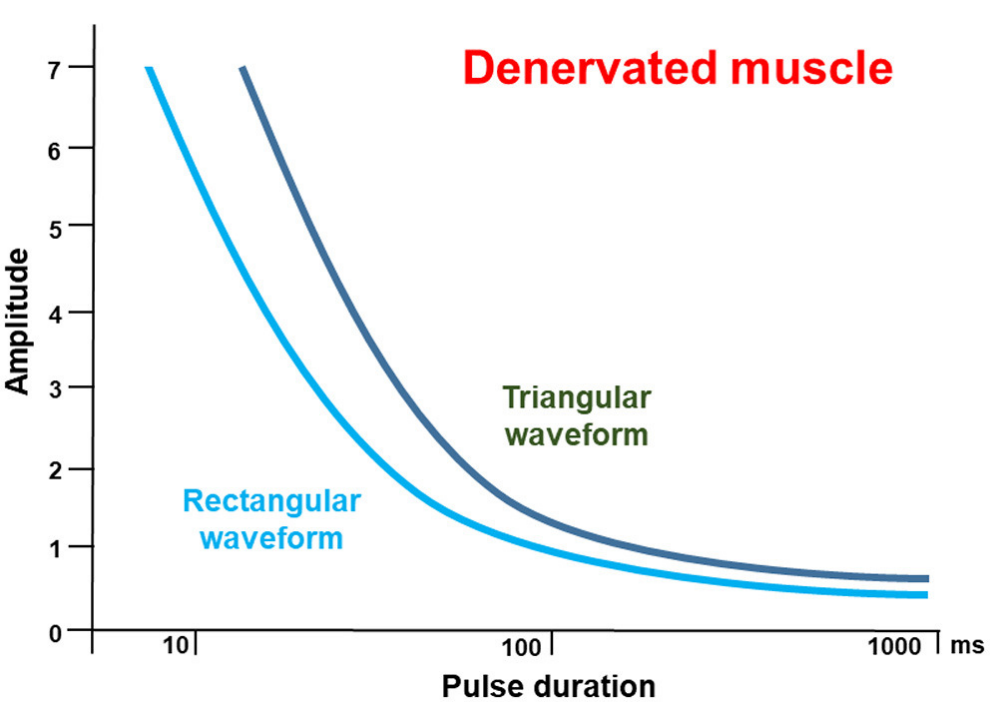
Pulse duration / threshold (I/T) curves only for denervated muscles for rectangular pulses (light blue) compared to triangular pulses (dark green) at higher magnification. The curve for rectangular pulses runs below the curve for triangular pulses up to a duration of 100 ms. In the area interest, above 100 ms, the curves run almost parallel. This is important, since triangular pulses are more effective than rectangular waveform, mostly because of the lower discomfort threshold. The idealized sketch follows the data from Arnold et al. (22).

According to the strength-duration curve of conventional rectangular pulses (Figure 1), the stimulation intensity needed for nerve or muscle activation exponentially decrease with the pulse duration until reaching a plateau (also known as rheobase). Strength-duration curves for stimulation of denervated muscles are different between rectangular pulses and triangular pulses (Figure 2). When using slow-rising triangular (or exponential) pulse shapes, the amplitudes needed for activation of denervated muscle fibers) require pulse durations longer than 10 ms (cf. Figures 1, 2). Using such parameters, the curve for nerves and innervated muscles is not decreasing anymore, but is increasing again, hence higher amplitudes are needed. For denervated muscle fibers this curve is shifted to longer pulse durations and is decreasing for longer pulse durations still. This strategy opens a window (shaded in Figure 1) for selective electrical stimulation, below the threshold for activating innervated muscles and nerve fibers directly, including the pain fibers in the skin. Therefore, the mean amplitude needed for triggering pain or discomfort is decreasing according to the strength/duration-curve up to pulse durations around 25 ms but is increasing again for longer pulse durations. This electrophysiological behavior is essential to understand the results of some studies that applied non-suitable parameters, as well as to choose an adequate electrical stimulation device, since only specialized devices allow such type of parameters.

## Effects of Facial Nerve and Muscle Stimulation Starting in the Acute Phase of Facial Palsy

**Figure 3:**
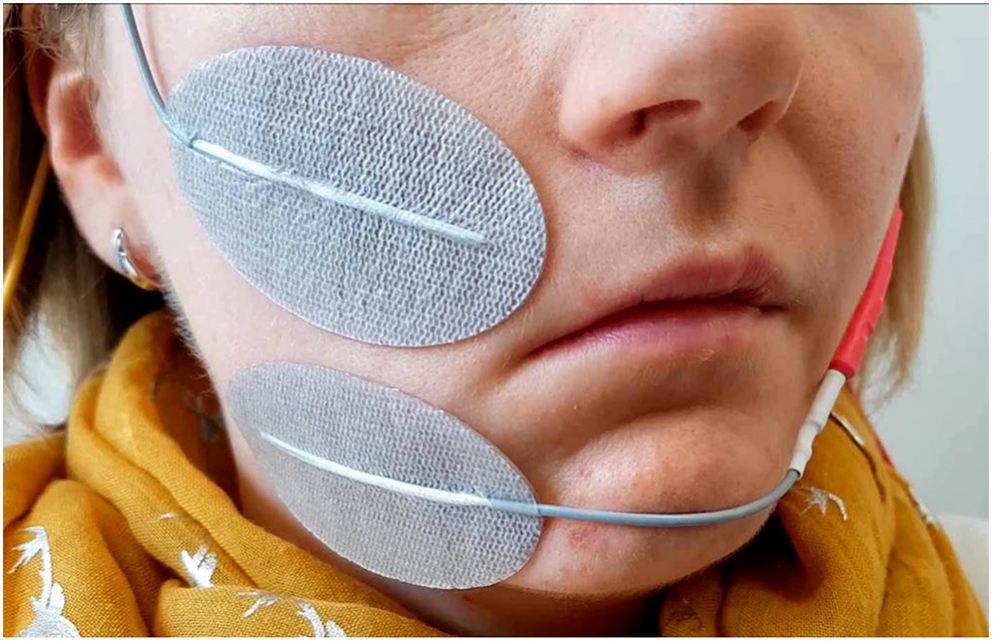
Optimal placement of the surface electrodes for transcutaneous facial muscle stimulation, here shown for the zygomatic muscle.

Effective facial muscle stimulation starts with optimal placement of the electrodes. An example for typical stimulation sites in the face is shown in Figure 3. It is an advantage that the facial muscles are lying directly superficial under the skin. The only adjacent muscles are the masseter and the temporal muscles. Unwanted stimulation of these chewing muscles can evoke discomfort and has to be avoided. On the other hand, facial muscles have the unique property that they are interwoven to each other and are partly overlapping (48). Hence, selective electrical stimulation of facial muscle has mainly to avoid unwanted stimulation of other neighboring facial muscles. The other important limitation is facial discomfort. Pain is sensed via the unaffected trigeminal nerve. The discomfort increases with the stimulus amplitude and pulse duration. It becomes painful and even intolerable with high amplitudes (49, 50). Unwanted stimulations and pain provide a confining framework for effective electrical stimulation without relevant side effects.

Table 3 summarizes several studies analyzing the effect of transcutaneous stimulation in the early acute phase of facial palsy. All studies shown in Table 3 only included patients with denervated muscles. Hence, an electrical stimulation of the facial nerve and its branches were not feasible. Instead, the electrodes had to be placed directly on the skin overlying the facial muscle of interest. Electrical stimulation was used to directly stimulate the facial muscles. The studies were examined for the pathophysiological findings and the framework of stimulation limitations. At least, two studies fully fulfill these criteria. Tuncay et al. addressed patients with Bell's palsy independently from the severity of the disease, i.e. half of the patients showed electrophysiological signs of facial nerve degeneration and the other half did not (56). The patients were randomized to receive electrical stimulation or not, beginning in the fourth week of acute palsy for 3 weeks with monophasic pulses of 100 ms, a pulse interval of 300 ms, at a rate of 2.5 Hz, until muscle contraction was visible. At 3 months later, the Facial Disability Index scores were improved similarly in both groups. House-Brackmann scale grading revealed greater improvement in the group with electrical stimulation. Electrophysiological assessments were performed of the frontalis and orbicularis oris muscles. The mean motor nerve latencies and compound muscle action potential amplitudes of both facial muscles were statistically shorter after electrical stimulation. Puls et al. compared patients with spontaneous regeneration without and with biphasic triangular electrical stimulation after typical etiologies (for instance, postsurgical, idiopathic, infectious) for severe degenerative facial nerve lesion (52). On average, an amplitude of 14 mA (range 6–20) and a pulse duration of 110 ms (range 100–280) was used. The amplitude and the phase duration were set individually for each patient based on visual inspection of the contraction of the zygomaticus muscle, depressor anguli oris muscle, and depressor labii muscle. The patients were advised to perform the home training twice per day for 10 min, 5 days per week. It should be highlighted that the follow-up was at least 1 year during the nerve regeneration. Outcomes were quantified using standardized grading systems. According to this grading, synkinesis was significantly lower with than without electrical stimulation (52). In a recent small but long prospective observational study, Arnold et al. also included three patients with early onset (15 days to 3.5 months) of electrical stimulation of the zygomatic muscle as home training after facial nerve lesion during schwannoma surgery with spontaneous regeneration or after facial nerve repair (22). Selective zygomatic muscle response in absence of discomfort and unselective contraction of other facial muscle was reproducibly obtained. A correct electrode placement in the target muscle area was very important. The most effective results were obtained with a pulse duration of 50 ms. The required amplitude was remarkably lower with ≤ 5 mA in these patients early after onset of the facial nerve lesion, compared to other patients with long-term (4 months to 16 years) denervation (where amplitudes up to 15 mA were needed). Rectangular pulses have a higher energy per pulse than the triangular pulses, where the pulse amplitude is rising till it reaches the peak, while for the rectangular pulse shape the amplitude jumps from zero to peak almost instantaneously (cf. Figure 2). Triangular pulse waveform was more effective than rectangular waveform (22), since the triangular pulse shape comes along with less pain and less unwanted co-contraction of other muscles.

**Table 3:** Important studies on electrical stimulation in the early acute phase of facial nerve lesion and muscle denervation.

**Study**	**Sample**	**Design**	**Setting**	Device*	**Pulse form**	**Inter-impulse gap**	**Phase duration**	**Amplitude**	**Training frequency**	**Training start after onset**	**Duration**	**Main outcome measures**	**Outcome**
Arnold et al. 2021 (22)	3 of 5 with acute palsy	Pro	Facial nerve reconstruction	STMISOLA or Stimulette r2x	triangular and rectangular biphasic	n.a.	150 ms (variable), also bursts at 7 Hz with a PW of 50 ms; single pulses	0.3–4 mA	session of 30 min	n. s.	6–12 months	Visible upward movement of the corner the mouth in video	Establishment of an effective and tolerable protocol
Sommerauer et al. 2020 (51)	1	Case report	Facial nerve reconstruction	Paresestim	n. s.	n. s.	up to 100 ms	up to 10 mA	2 times 10 min per day	n. s.	19 months	Visible facial movements	Improvement to bridge reinnervation time
Puls et al. 2020 (52)	13	Retro	spontaneous reinnervation after postop paralysis	Paresestim	biphasic triangular	Same length as phase duration	110 ms (range 100–280); single pulses	14 mA (range 6–20)	twice per day for 10 min, 5 days per week.	4.7–13.2 days	13–37 months	Improvement in eFACE and Sunnybrook grading	Lower synkinesis rate
Mäkelä et al. 2020 (53)	15	Pro?	Acute facial palsy	Self-made stimulator	biphasic	200 ms	0.4 ms	average 4.9 mA (range: 3–8)	2 h	7–82 days	at 2 days	Dry Eye Questionnaires; NRS for pain/discomfort	Producing a reliable blink
							250 Hz						
Loyo et al. 2020 (54)	planned	RCT	Bell's palsy	OrthoStim	monophasic	30 s	100 μs		10 contractions over 20 min	<30 days	6 months?	eFACE, House-Brackmann, Sunnybrook, FaCE, SAQ	ES promotes recovery
							35 Hz						
Kim and Choi 2016 (55)	30 w ES	Pro RCT	Bell's palsy	version 3; Kwangwoo Medix	rectangular, monophasic	80 μs	10 ms	average 1.4 mA	Sessions: “continuous”; Sessions per week: “continuous”	2 weeks	2 months	House-Brackmann; Sunnybrook	Better than without ES
	30 w/o ES						20 Hz						
Tuncay et al. 2015 (56)	(28 w ES)	Pro RCT	Bell's palsy	Dynatron 438	monophasic	300 ms	100 ms	n. s.	5 days per week three sets of 30 minimal contractions	n. s.	3 weeks	House-Brackmann; FDI	Improved functional facial movements
	32 w/o ES						pulse rate of 2.5 pulses/sec						
Frigerio et al. 2015 (57)	40	Pro	Acute facial paralysis	STMISOLA	?	1 ms	0.4–1 ms	7.2 mA (1–15)	?	6-58 days	once	High-speed video analysis of blink characteristics	Producing a reliable eye closure
							100-50 Hz						
Alakram and Puckree 2010 (58)	16	Pro RCT	Bell's palsy	EV-803 Digital SD TENS	biphasic	10 μs	10 μs	n. s.	30 min. Sessions per week: 1	n. s.	3 months	FDI	No effect on recovery rate
							10 Hz						
Mosforth and Taverner 1958 (59)	83	Pro RCT	Bell's palsy	Ritchie-Sneath	galvanic	n. s.	100 ms	n. s.	3 sets of 30 contractions, Sessions per week: 3	<2 weeks	up to 12 months	Clinical examination	No effect on recovery rate

ES, electrical stimulation; w, with; w/o, without; pro, prospective; retro, retrospective; RCT, randomized controlled trial; n.a., not applicable; n.s., not specified, ^*^Devices used: Paresestim (Krauth + Timmermann, Hamburg, Germany), PierenStimParese (Schwa-Medico, Ehringshausen, Germany), Stimulette r2x (Dr. Schufried, Vienna, Austria), Dynatron 438 device (Enraf, Germany); EU2F Eutrophic Stimulator (NeuroTech Limited); version 3; Kwangwoo Medix, Inc., Seoul, Korea; Nervepace S-200 system (Neumed Inc, Lawrenceville, NJ); STMISOLA (BIOPAC, Goleta); OrthoStim (VQ Ortho CareVR, Vista). FaCE, Facial Clinimetric Evaluation (FaCE) Scale; FDI, Facial Disability Index; eFACE, Electronic Facial Paralysis Assessment; Sunnybrook, Sunnybrook Facial Grading System; House-Brackmann, House-Brackmann grading scale; NRS, numeric rating scale.

Electrostimulation was used in one patient in Arnold et al. to bridge the waiting time until a reconstruction of the facial nerve via a hypoglossal-facial jump nerve suture was performed 8 months later. In the first months after nerve lesion, electrodiagnostics might not be clear regarding the fate of the facial nerve (6). Later on, beyond 6 months after onset, a denervation without probability of recovery becomes clearly obvious. Electrostimulation can maintain the muscle tone before decision making for or against a nerve reconstruction is completed (51).

In order to protect the eye, electrostimulation to elicit blinking was first studied by Frigerio et al. in patients with acute facial paralysis and onset of <6 weeks (57). An average current of 4.6 mA, average pulse width of 0.7 ms, 100–150 Hz, were needed to induce an eye twitch. Complete eye closure requires an average of 7.2 mA, but it was only achieved in about half of the patients. These parameters were tolerable for most patients. A comparable setting was used by Makela et al. (53). They included patients <3 months after onset of Bell's palsy. Biphasic stimulation, pulse duration of 0.4 ms, and a 250 Hz pulse repetition frequency, and a pulse train duration of 200 ms were used. With this setting and an average current of 4.9 mA, they could also produce a blink in about half of the patients. The parameters used in these two electrically induced blink studies were derived from prior studies in healthy probands, i.e. persons with normal facial muscles. Furthermore, Makela et al. found out that the blink stimulation worked in patients with less severe palsy and faster recovery, i.e. patients with neurapraxia or mild axonotmesis. Both blink studies can be interpreted in such a way that very short pulse durations (<1 ms) are able to stimulate intact muscle fibers but not denervated muscles fibers. The studies from Puls et al. or Arnold et al. clearly show that short-term denervated facial muscles fibers need pulse duration of about 50–200 ms to be effective and tolerable (22, 52).

These observations are important when interpreting the published results and clinical protocols: Loyo et al., for example, published a protocol planning to use 100 μs pulses at 35 Hz stimulation rate in a prospective, double-blinded, randomized, placebo-controlled study investigating the effect of electrostimulation on recovery from acute Bell's palsy in patients with poor prognostic factors (54). We are concerned that this might not be effective, especially when applied to facial muscles of patients with poor prognosis, i.e. with high degree of denervation. Moreover, Alakram and Puckree preformed an electrostimulation study concluding that stimulation was not effective to improve functional recovery after Bell's palsy (58). We believe, in contrast, that this was likely due to the short duration pulses (10 μs) they used when attempting to stimulate denervated muscles (60). If such important technical aspects are neglected when selecting studies for a systematic review on the topic, it is not surprising if the conclusion is that there is no evidence to support the use of electrical stimulation during the acute phase of recovery after Bell's palsy (61). Mosforth and Taverner used in one of the first studies in the field a longer pulse duration of 100 ms (59). They observed in some Bell's palsy patients with severe denervation of the facial musculature that the muscles tired quickly, and that the number of contractions was therefore reduced. Overall, they saw no effect, but standardized outcome measures were not used in this older study. Recently, Kim and Choi performed a randomized study for cases of mild-to-moderate grade Bell's palsy (55). The patients received standard drug treatment with/without electrostimulation. Electrostimulation was performed with a pulse duration of 10 ms, 20 Hz frequency, with sub-threshold pulses (average 1.4 mA). The rationale for this unconventional sub-threshold design was related to a prior successful experimental study also using only sub-threshold pulses. Certainly, the use of the sub-threshold pulses avoids side effects but any effects on denervated muscle fibers remain doubtful. The electrostimulation group showed a higher overall rate of recovery.

## Effects of Laryngeal Nerve and Muscle Stimulation in the Acute Phase of Recurrent Laryngeal Nerve Palsy

**Figure 4:**
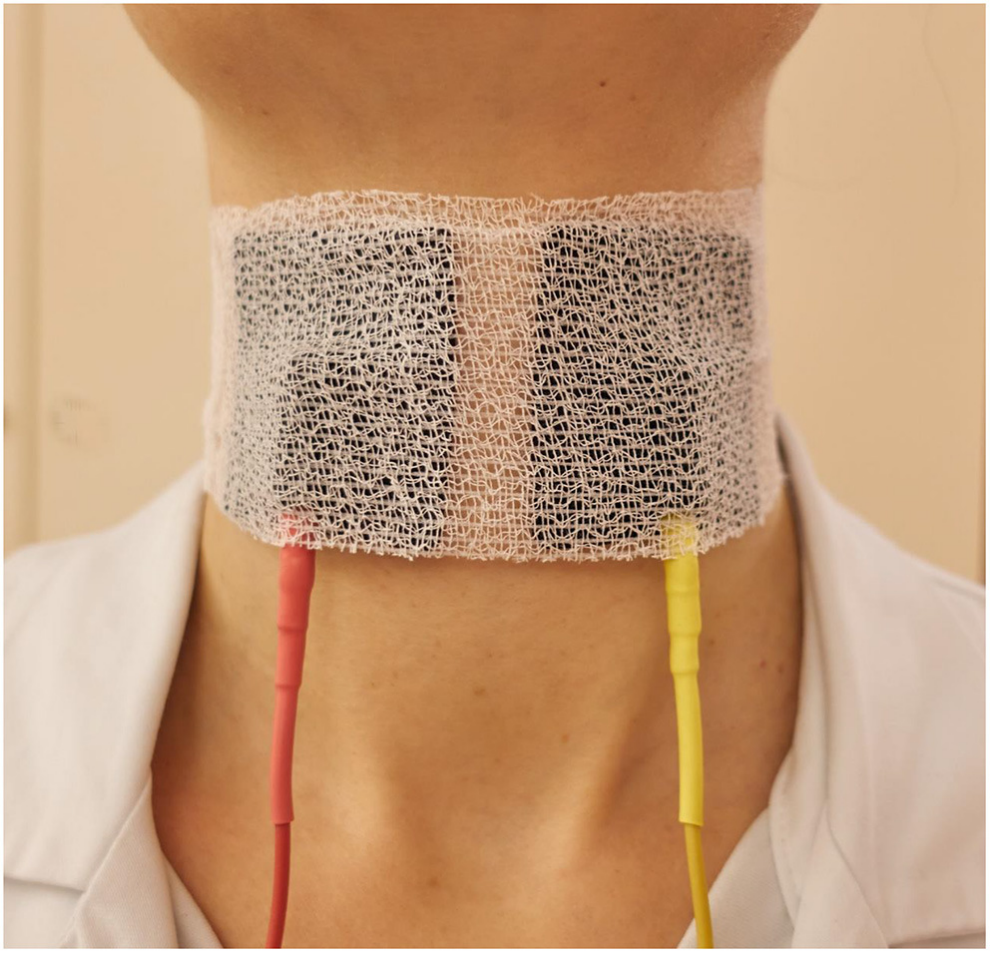
Optimal placement of the surface electrodes for transcutaneous intralaryngeal muscle stimulation. The electrodes should be additionally fixed by a circular cervical dressing.

Beyond pain as a general limitation of electrical stimulation, it has to be considered that the intrinsic laryngeal muscles and their innervation are lying deep in the neck. They are covered by the laryngeal cartilages and several other more superficial, but also other throat muscles. This has two important consequences: Unlike in the face, where the facial muscles lie directly under the skin, unintended stimulation of muscles lying more superficial than the laryngeal muscles (for instance, sternohyoid muscle, sternothyroid muscle, or platysma) has to be avoided. Secondly, as it is known from electrostimulation studies to induce swallowing, very short pulses (<1 ms) are too short to reach the deeper lying laryngeal muscles (62). Typical side effects that have to be avoided during optimal stimulation of the laryngeal muscles are cough, swallowing, and discomfort. An example for typical stimulation sites for recurrent laryngeal nerve stimulation in the neck is shown in Figure 4. The correct placement of the electrodes is very important. If the electrodes are placed more in direction of the area between the hyoid bone and the thyroid cartilage, it might be that the internal superior laryngeal nerve is stimulated, but not the internal laryngeal muscles (36).

Several studies have analyzed the effect of transcutaneous stimulation in the early acute phase of recurrent laryngeal nerve palsy (Table 4). All cited studies included patients with diagnosed denervated vocal folds exclusively. Therefore, the electrodes were placed on the skin overlying the thyroid cartilage for the electrical signal to transfer the thyroid cartilage and to stimulate the denervated vocal folds on the inner/medial side of the thyroid cartilage. Like for facial muscles stimulation, the effectiveness of these studies has to be evaluated under the light of the limits of electrical stimulation given by these side effects. Schleier and Streubel were the first to present a series of systematic electrical stimulation for different laryngeal diseases (69). Patients with dysphonia (i. e. normally innervated laryngeal muscles) and patients with recurrent nerve lesions and very variable denervation time (i.e. variable status of denervated and reinnervated laryngeal muscles) all received the same electrical stimulation: They used a standard setting with pulse length of 1 ms independent of the innervation status of the laryngeal muscles. Based on subjective evaluation of the voice, they also reported improvements for the patients with vocal fold paralysis, although these stimulation parameters are not able to stimulate denervated laryngeal muscles. Several research groups recognized that denervated muscles need longer pulse lengths for stimulation but can be stimulated at lower currents than normal laryngeal muscles (67). Kruse recommended a pulse length of 50 ms for weakened laryngeal muscles (i.e., partially innervated/reinnervated) and 250 ms for paralyzed muscles (i.e., denervated muscles). Ptok and Strack performed the first prospective trial directly comparing voice exercise therapy ± electrical stimulation in patients with unilateral vocal fold paralysis between 2 weeks and 6 months after onset, i.e. they might have not only included patients in the denervated but also some patients with partially reinnervated status (66). They used a long pulse duration of 240 ms and a current at suprathreshold (amplitude was adjusted to a just-visible twitch). After a therapy period of 3 months, vocal fold irregularity improved to a significantly greater extent in the electrostimulation group. Maximum phonation time increased similarly in both groups. In contrast, Perez Garcia et al. investigated the effect of electrical stimulation in patients with unilateral vocal fold paralysis between 10 and 24 months after onset, i.e. in late denervation and dominantly reinnervation phase (65). Laryngeal electromyography was not performed. It is most likely, that most of the patients were included in a reinnervated stage of the disease. This would explain why pulse duration of 0.1 to 0.5 ms and currents of 0 to 10 mA were effective to evoke vocal fold movements seen by laryngoscopy. Acoustic analysis revealed significant improvements. The applied parameters would not have been effective for patients early after onset in the denervation phase of the disease. Like the before mentioned study, also Garcia Perez et al. did not pay attention on safety issues or systematically investigate side-effects (65).

**Table 4:** Important studies on electrical stimulation in the early acute phase of recurrent laryngeal nerve lesion and muscle denervation.

**Study**	**Sample**	**Design**	**Setting**	Device^*^	**Pulse form**	**Inter-impulse gap**	**Phase duration**	**Amplitude**	**Training frequency**	**Training start after onset**	**Duration**	**Main outcome measures**	**Outcome**
Kurz et al. 2021 (63)	25 w ES	Retro	UVFP after thyroid surgery	Stimulette rx or Stimulette r2x	Biphasic	50 ms	100–250 ms; single pulses	n.s	twice a day for 25 min (3 × 5 min; stimulation interval with 2 × 5 min breaks) i		up to 3 months	RBH, vocal fold position, glottal closure during phonation	As effective as voice therapy
	26 w/o ES												
Kurz et al. 2021 (64)	32	Pro	UVFP	STMISOLA	Biphasic triangular	n.s	1, 10, 25, 50, 100, 250, and 500 ms	1–20 mA	Trains of 5 pulses	n.a.	once	Sensitivity and discomfort threshold, vocal fold adduction at rest/phonation	Establishment of an effective and tolerable protocol
Garcia Perez et al. 2014 (65)	10	Pro	UVFP	PIC16F877A, Microchip Technology		n.s	•−0.5 ms;	0–10 mA	n.s	10 – 24 months	10 weekly 30-min-long stimulation sessions	F0, jitter (%), shimmer (%), HNR; NNE; MPT	Acoustic analysis revealed significant improvements
							500 Hz						
Ptok and Strack. 2008 (66)	33 w ES	RCT	UVFP after iatrogenic or idiopathic lesion	VocaSTIM, Physiomed	Biphasic	n.a.	240 ms;	n.s	n.s	>2 weeks; <6 months	3 months	standardized text, MPT, CFx, CFx index	More effective than voice therapy
	36 w/o ES						single pulses						
Kruse 1989 (67)	Review	Retro	UVFP after thyroid surgery	Laryngoton	Biphasic	n.a.	50–250 ms;	<20 mA	n.s	n.s		pitch change at constant phonation	More effective than voice therapy
							single pulses						
Dahl and Witt 2006 (68)	18 w ES	Pro	UVFP after iatrogenic or idiopathic lesion	VocaSTIM, Physiomed	Biphasic	n.a.	240 ms;	n.s	10x, 2–3/week	n.s	16–20 weeks	PPQ; APQ; SPI; NHR; Jitter, Shimmer; VTI; glottal gap during phonation	More effective than voice therapy
	8 w/o 8						single pulses						
Schleier & Streubel 1980 (69)	42 w ES	Retro	several etiologies, *N* = 46 with UVFP	RS 8 & RS 12 TUR	Biphasic	0	1.5–2 ms;	<6 mA	n.s	n.s	10 min, 21 sessions	vocal fold position	More effective than voice therapy
	4 w/o ES						120–280 Hz						

*ES, electrical stimulation; w, with; w/o, without; UVFP, unilateral vocal fold palsy; pro, prospective; retro, retrospective; RCT, randomized controlled trial; n.a., not applicable,; n.s., not specified*;

* *Devices used: Stimulette rx or Stimulette r2x (Dr. Schufried, Vienna, Austria), STMISOLA (BIOPAC, Germany), Microchip technology (Arizona, United States), VocaSTIM (Physiomed, Schnaittach, Germany), Laryngoton (IPS, Braunschweig, Germany), RS 8 & RS 12 TUR (Starkstrom Anlagenbau VEB, former East Germany); HNR, harmonics-to-noise ratio; NNe, normalized noise energy (NNE); MPT, maximum phonation time; CFx, vocal fold irregularity; PPQ, pitch perturbation quotient; APQ, amplitude perturbation quotient; SPI, soft Phonation Index; NHR, noise-to-harmonic ratio; VTI, voice turbulence index*.

**Figure 5:**
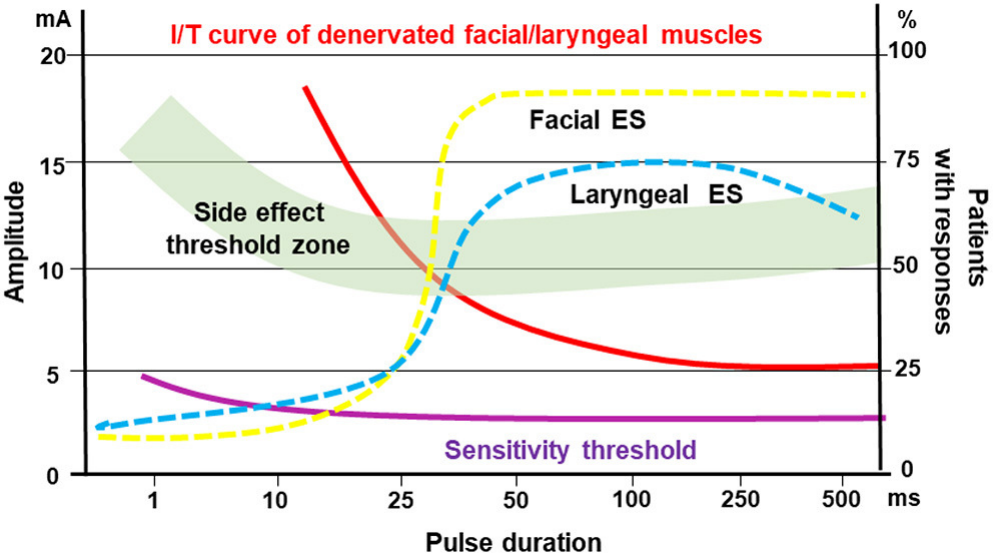
Idealistic view on the optimal window for electrical stimulation of early acute denervated facial or laryngeal muscles. Pulse duration / threshold (I/T) curve (red) for denervated facial and laryngeal muscles in relation to side effect threshold (i.e. discomfort, pain, stimulation of other muscles; green zone). Sensitivity threshold (purple) illustrates the threshold when the patients feel the electrical stimulation (all left Y-axis). The dotted lines show the efficacy of facial muscle (yellow) or laryngeal muscle (blue) stimulation in % of patients with responses (right Y-axis).

From the functional point of view it is very important to define the innervation status of the patients, i.e. with laryngeal EMG, and to prove that the electrical stimulation de facto leads to vocal fold closure, i.e. with a laryngoscopy. These prerequisites were considered by Kurz et al. (63). First, they retrospectively compared standard voice therapy versus home training with electrical stimulation in patients with early unilateral vocal fold paralysis. Using single pulses with a duration of 100–250 ms it was possible to selectively stimulate the otherwise immobile vocal folds. Functional outcome and recovery rate were similar in both groups. Hence, like seen for the facial nerve, correct electrical stimulation does not hinder the regeneration of the recurrent laryngeal nerve. These results consequently led Kurz et al. to a prospective trial to systematically analyze optimal parameters for effective and tolerable stimulation of the vocal folds (64). Most effective were pulse durations between 50 and 100 ms with an average amplitude of 7.1 mA. These parameters were also optimal to reduce the rate of side-effects like discomfort, co-contraction of extralaryngeal muscles, cough or undesirable swallowing reflex (cf. Figures 1, 5).

## Recommendations for Optimal Electrical Stimulation Early After Onset of Facial Nerve or Recurrent Laryngeal Nerve Palsy

**Figure 6:**
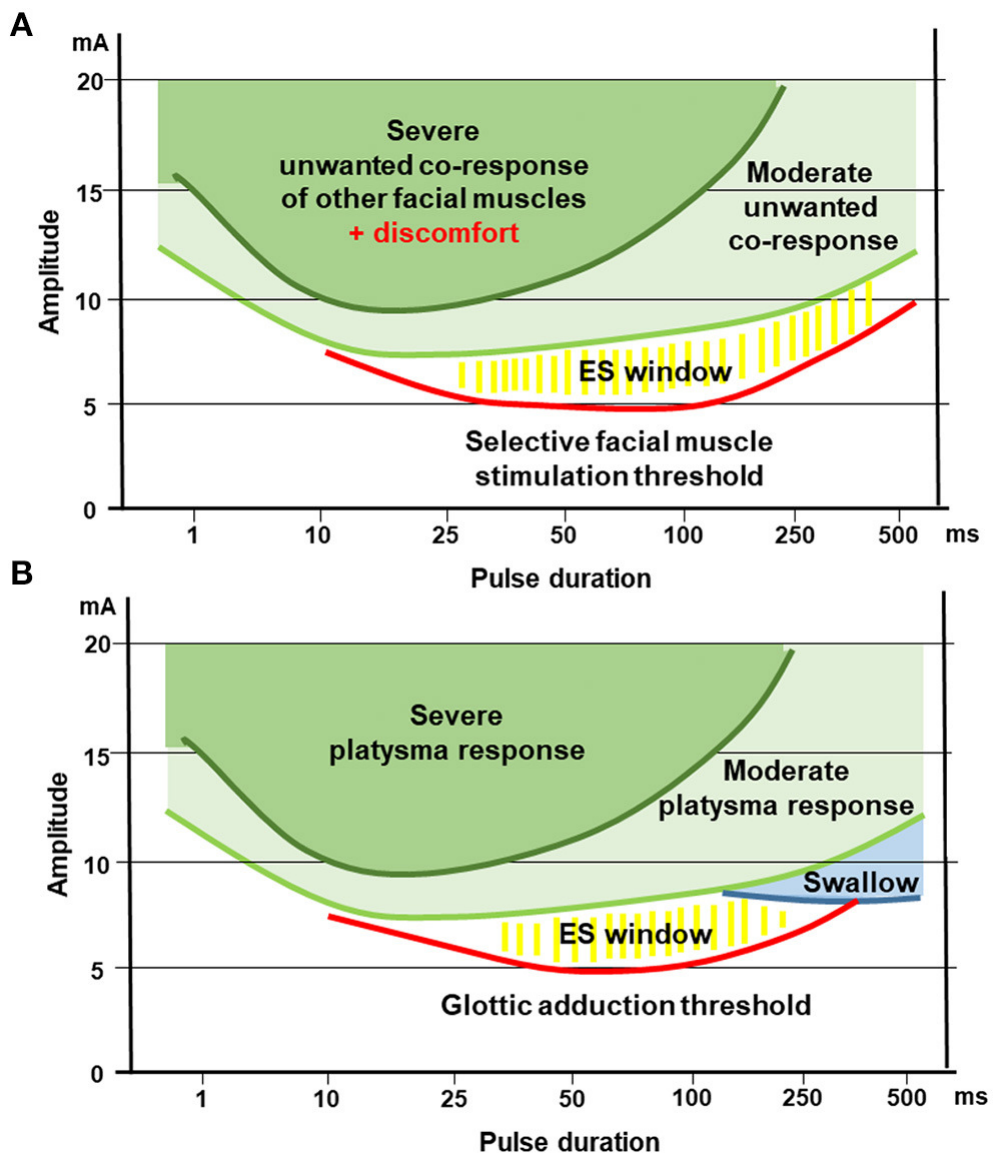
Optimal window for electrical stimulation of early acute denervated facial or laryngeal muscles, relation to specific side effects highlighted. This figures illustrates the similarities for optimal electrostimulation of the facial or laryngeal muscle stimulation. **(A)** Stimulation of facial muscles. **(B)** Stimulation of intralaryngeal muscles for glottic adduction.

Based on the presented results, we see similarities between optimal stimulation parameters for the facial and the recurrent laryngeal nerve in the early acute phase of muscle denervation. Figures 5, 6 are summarizing the optimal window of an efficient electrical stimulation of denervated facial or laryngeal muscles. Stimulation of early denervated facial muscles is possible with rectangular pulses with durations below 10 ms, however, it rapidly leads to undesired side effects as higher currents are needed in comparison with longer pulse durations. The same holds true for the larynx: Stimulations of the vocal folds conducted with pulse widths below 10 ms are likely to be ineffective since they are likely to cause a moderate to severe platysma before being able to effectively cause vocal fold medialization. In the early phase of facial palsy, electrical stimulation of the denervated facial muscles seems to be optimal with pulse lengths of 50–200 ms. Most of the published reports focus on the zygomatic muscle (22, 52). Some data have been published for frontalis, corrugator supercilii, palpebral part of orbicularis oculi, levator labii superioris alaeque nasi, levator labii superioris, levator anguli oris, risorius, orbicularis oris, depressor anguli oris, depressor, labii inferioris, and levator menti muscles (56). Stimulations conducted at 50 and 100 ms are optimal for the vocal folds as such stimulation is more likely to elicit medialization and voice change without side effects. Stimulations conducted at 250 and 500 ms are too long for both, the facial and laryngeal muscles. At these pulse duration levels, more than half of the patients report discomfort. For the larynx, it is more likely to elicit swallow reflex with medialization and voice change. The first parameter to be defined is the pulse duration, since it is more limited by the side effects. Then, an effective stimulation intensity should be chosen to elicit responses for the given pulse duration. Furthermore, the possible frequency range is limited also by the pulse duration and the minimum pause between pulses. For example, for a duration of the cathodic phase of 100 ms, the same duration of 100 ms is needed for the anodic phase of the pulse for charge balance. Many stimulation devices are then not allowing a pause in-between pulses shorter than the chosen stimulation phase. In the given example, the duration between the start of one stimulation pulse till the start of the next stimulation pulse is 100 ms +100 ms +100 ms = 300 ms. Hence, the maximal frequency of electrical stimulation would be 3.3 Hz (frequency = 1/ (sum of pulse duration) = 1/300). We recommend using triangular pulses instead of rectangular pulses both for the face and the larynx. This pulse shape goes along with less pain in the skin and reduced non-specific co-contraction of unwanted other muscles in the neighborhood of the target muscles

## Conclusions and Future Prospects

The pathophysiology of facial nerve and recurrent nerve lesion have much in common. Degenerative lesion of either nerve causes a complex damage of interactive or even antagonistic muscles. Both lesions and the consequences for non-verbal and verbal communication have a major impact on quality of life of the patients. The affected target muscles are accessible for electrical stimulation early after onset of the lesion in most patients. The major reservation against a widespread use of electrical stimulation for treatment of unilateral facial or vocal fold paralysis was the notion that it is difficult to determine or even impossible to find a combination of parameters capable to generate a selective stimulation delivered by surface electrodes. This is clearly obsolete. Applying the recent concepts of electrostimulation of denervated muscles early after onset of facial nerve or recurrent nerve lesion in combination with the correct electrostimulation devices and electrodes allow a safe and selective electrostimulation. Correctly applied, electrical stimulation does not hinder the regeneration of the facial or recurrent laryngeal nerve. It maintains muscle function in the time between onset of the lesion, and spontaneous reinnervation or surgically induced reinnervation of the target muscles. It may also reduce synkinetic reinnervation, although data are still rare to sparse to allow general conclusions. The afferent feedback is activated by the stimulation of the muscles, too. This may help to keep the cortical representation of the motor patterns intact. So by stimulation denervated muscles, the broken feedback loop controlling the high precision movements of the face and larynx could be temporary closed. This is of great importance because then early onset electrical stimulation would not only be of interest to reduce denervation atrophy and to bridge the regeneration phase mainly to maintain muscle volume. It would additionally be of interest to improve functional recovery and to reduce adjuvant rehabilitation efforts.

More quality randomized control trials are needed, using internationally recognized outcome measures, consideration of potential safety concerns in study design, standardized electrical stimulation programs and consistent follow-up (61). An objective and systematic recording of complications is also mandatory (61). Important open research questions are for the facial paralysis to determine the extent by which external stimulation is able to effectively improve eye closure, closure of the mouth while drinking or eating, and non-verbal communication abilities. it is equally important to determine optimal stimulation for best voice quality. It has to be studied systematically, if fixed parameters should be used, or if the stimulation protocol should be adapted to the degeneration/regeneration status of the patient. Still in the phase of denervation, it might be that weaker currents are needed once the denervated muscles are adapted to the electrical stimulation (70). At a certain stage, the patient will present a mixed situation out of still denervated and already innervated muscle fibers. It might be of interest to combine stimulation protocols to stimulate both the denervated and reinnervated muscle fibers. Finally, an open field is the intensity of the treatment. The more sessions and time are needed for a home-based training or training with a therapy, this might decrease the compliance of the patients. If patients see a therapy advantage, the high number of therapeutic electrostimulation sessions serves as an indicator of good compliance for this kind of therapy, like in the study by Ptok and Strack (66).

When we get more data on optimal electrostimulation in the early phase of facial or recurrent laryngeal nerve stimulation, this knowledge will also help us in the long run, to adapt the concept to permanent and implantable electrostimulation of irreversibly denervated muscles. So far, a first human implantable system has been developed for patients with bilateral vocal fold paralysis and synkinetic reinnervation, i.e. a system for nerve stimulation in patients with reinnervated laryngeal muscles (71). We are not yet there for the face, but the clinical feasibility has at least been shown also for the treatment of post-paralytic synkinesis by extrinsic stimulation with a system intended for implantation (72).

## Author Contributions

AK and GV: literature research, investigation, and editing. DA: investigation. BS-S: conceptualization, validation, supervision, and project administration. WM: conceptualization, and validation. OG-L: conceptualization, methodology, validation, visualization, writing-original draft and writing-review and editing, supervision, and project administration. All authors contributed to the article and approved the submitted version.

## Conflict of Interest

The authors declare that the research was conducted in the absence of any commercial or financial relationships that could be construed as a potential conflict of interest.

## Publisher's Note

All claims expressed in this article are solely those of the authors and do not necessarily represent those of their affiliated organizations, or those of the publisher, the editors and the reviewers. Any product that may be evaluated in this article, or claim that may be made by its manufacturer, is not guaranteed or endorsed by the publisher.
